# Identification and functional analysis of ovarian lncRNAs during different egg laying periods in Taihe Black-Bone Chickens

**DOI:** 10.3389/fphys.2024.1358682

**Published:** 2024-02-15

**Authors:** Yunyan Huang, Shibao Li, Yuting Tan, Chunhui Xu, Xuan Huang, Zhaozheng Yin

**Affiliations:** College of Animal Science, Zhejiang University, Hangzhou, China

**Keywords:** long non-coding RNA, Taihe Black-Bone Chicken, ovary, egg production, RNA-seq

## Abstract

**Introduction:** Long non-coding RNA (lncRNA) refers to a category of non-coding RNA molecules exceeding 200 nucleotides in length, which exerts a regulatory role in the context of ovarian development. There is a paucity of research examining the involvement of lncRNA in the regulation of ovary development in Taihe Black-Bone Chickens. In order to further investigate the egg laying regulation mechanisms of Taihe Black-Bone Chickens at different periods, transcriptome analysis was conducted on the ovarian tissues at different laying periods.

**Methods:** This study randomly selected ovarian tissues from 12 chickens for RNA-seq. Four chickens were selected for each period, including the early laying period (102 days, Pre), the peak laying period (203 days, Peak), and the late laying period (394 days, Late). Based on our previous study of mRNA expression profiles in the same ovarian tissue, we identified three differentially expressed lncRNAs (DE lncRNAs) at different periods and searched for their cis- and trans-target genes to draw an lncRNA-mRNA network.

**Results and discussion:** In three groups of ovarian tissues, we identified 136 DE lncRNAs, with 8 showing specific expression during the early laying period, 10 showing specific expression during the peak laying period, and 4 showing specific expression during the late laying period. The lncRNA-mRNA network revealed 16 pairs of lncRNA-target genes associated with 7 DE lncRNAs, and these 14 target genes were involved in the regulation of reproductive traits. Furthermore, these reproductive-related target genes were primarily associated with signaling pathways related to follicle and ovary development in Taihe Black-Bone Chickens, including cytokine-cytokine receptor interaction, TGF-beta signaling pathway, tyrosine metabolism, ECM-receptor interaction, focal adhesion, neuroactive ligand-receptor interaction, and cell adhesion molecules (CAMs). This study offers valuable insights for a comprehensive understanding of the influence of lncRNAs on poultry reproductive traits.

## 1 Introduction

Chickens are one of the primary domesticated poultry in China, serving as a significant source of meat and egg-related products (Zhang et al., 2022). In recent years, the improvement in living standards has increased the demand for chicken meat and eggs. However, current production levels often fall short of domestic demand (Iannotti et al., 2014). On the other hand, one of the primary goals in egg laying chicken breeding is achieving high egg production, which plays a crucial role in enhancing production efficiency. Currently, low egg production rates are also one of the key factors hindering the development of the poultry industry (Mu et al., 2021; Chomchuen et al., 2022).

The ovary is the primary organ within a hen’s body responsible for producing eggs. It contains numerous small follicles. A follicle consists of both the oocyte and follicular cells, including granulosa cells and theca cells. The interactions among these components form a complex system (Li et al., 2011). Follicle development represents the basic functional unit of the ovary and is a finely regulated process. Follicle development is influenced by both the hypothalamus-pituitary-gonad (HPG) axis and environmental factors. Within the HPG axis (Sun et al., 2021), various hormones are secreted, including follicle-stimulating hormone (FSH), luteinizing hormone (LH) and inhibin (Mishra et al., 2020). FSH’s primary role is to stimulate follicular development within the ovary, promoting follicle maturation and ultimately leading to oocyte development and ovulation (Ghanem and Johnson, 2019). LH induces ovulation by triggering the release of matured follicles from the ovary and promoting the formation of the corpus luteum to maintain its function. Inhibin, on the other hand, inhibits the secretion of FSH and LH from the pituitary gland, helping to maintain an appropriate number of follicles within the ovary. These hormones work in harmony, regulating the hen’s sexual maturation, follicular development, ovulation, and reproductive cycles (Zhao et al., 2018). Normal HPG axis function is of utmost importance for the reproductive health of chickens, while the health and normal function of the ovary are essential for hen’s reproduction and the production of high-quality eggs.

Long non-coding RNAs (lncRNAs) are a class of RNA molecules that typically exceed 200 nucleotides in length and unlike protein-coding RNAs, do not encode proteins. They have long been overlooked as transcripts with no apparent function (Bonasio and Shiekhattar, 2014). However, in recent years, with the advancement of next-generation sequencing technologies in the field of genetics, researchers have discovered that lncRNAs play significant biological roles in the ovary (Zhang et al., 2020). For instance, lncRNAs are involved in regulating the development and maturation of ovarian follicles, influencing the proliferation, differentiation, and apoptosis of follicular cells, thus impacting follicular growth and development (Zhao et al., 2020). LncRNAs also affect the quality of eggs by regulating chromatin structure and epigenetic modifications in oocytes, which are crucial for normal fertilization and embryo development (Minarovits et al., 2016). Furthermore, lncRNAs can modulate hormone sensitivity in the ovary, affecting signaling pathways such as FSH and LH, thereby influencing follicular development and ovulation. Some lncRNAs are associated with the occurrence and progression of ovarian cancer. They may play roles in cancer cell proliferation, invasion, and metastasis, making them potential biomarkers or therapeutic targets for cancer. Additionally, lncRNAs in the ovary may regulate immune responses, influencing ovarian inflammation and autoimmune ovarian diseases (Ren et al., 2015). Recently, it has been discovered that lncRNAs can promote granulosa cell apoptosis in ducks and participate in lncRNA-miRNA-mRNA co-expression networks, potentially affecting duck follicles (Wu et al., 2021). Whole-genome sequencing of ovarian tissues from different developmental stages of Hu sheep revealed that lncRNA target genes may be involved in follicle development, steroid hormone-mediated signaling pathways, steroid hormone biosynthesis, gonadotropin responses and insulin-like growth factor receptor binding (Shabbir et al., 2021). Using confocal transmission electron microscopy and RNA-Seq, Macaulay et al. (2014) discovered that granulosa cells surrounding bovine oocytes transport a significant amount of nutrients and substances, including mRNA and lncRNA. Brown et al. (2014), in their research on *Drosophila* ovaries, also found a substantial presence of promoter-associated antisense lncRNAs, which may regulate the transcriptional activation of their homologous genes, playing a crucial role in early embryonic development before implantation. Additionally, non-additive lncRNAs MSTRG.6475.20 and MSTRG17017.1, along with their non-additive target genes (*GNAQ*, *CACNA1C*, and *TGFB1*), were discovered in the molecular mechanisms of chicken hybrid advantage, involving the gonadotropin-releasing hormone (GnRH) signaling pathway and female gonad development (Wang et al., 2022).

Taihe Black-Bone Chicken, originating from Taihe County in Jiangxi Province, China, boasts a rich history of breeding. It is not only recognized in traditional medicine for its unique medicinal and culinary properties but also holds a high ornamental value, thus contributing significantly to its economic importance (Mi et al., 2018). However, Taihe Black-Bone Chickens have low egg production performance, and systematic breeding work started relatively late. The breeding potential has not yet been fully explored, reflecting the problem of insufficient production performance faced by local breeds. According to our previous work, 1,167 Taihe Black-Bone Chicken breeding core group hens were used as experimental subjects, and egg production data were systematically recorded. It was found that Taihe Black-Bone Chickens have a longer early laying period and lower egg production rate. They enter the peak laying period for a longer time, and the egg production rate reaches the highest at about 72.49% at 30 weeks of age. The egg production peak maintains a rate of 70% between 30 and 32 weeks of age, only 3 weeks, after which the egg production rate rapidly declines. In this study, we collected ovarian tissues from Taihe Black-Bone Chickens at three distinct laying periods: the early laying period (102 days, Pre), the peak laying periods (203 days, Peak) and the late laying period (394 days, Late). To identify differentially expressed lncRNAs (DE lncRNAs) across these periods, we conducted lncRNA sequencing on ovarian tissues. Additionally, we performed functional enrichment analysis using Gene Ontology (GO) and the Kyoto Encyclopedia of Genes and Genomes (KEGG) to uncover the regulatory roles of DE lncRNAs in ovarian development. These findings offer novel insights into enhancing the ovarian development of Taihe Black-Bone Chickens and provide new prospects for increasing their egg production rate.

## 2 Materials and methods

### 2.1 Ethics approval

Our study was carried out in compliance with the ARRIVE guidelines (AVMA Guidelines for the Euthanasia of Animals: 2020 Edition). All animal care and experimental procedures were approved by the Institutional Animal Care and Use Committee of Zhejiang University (protocol code ZJU14814 and 23 May 2022 of approval). All research work strictly adhered to the experimental animal welfare and ethical guidelines of Zhejiang University (ZJU).

### 2.2 Animal

Taihe Black-Bone Chickens were obtained from the Poultry Breeding Center of Jiangxi Taihe Livestock Company (Taihe county, Jiangxi province, China). All chickens were subjected to identical rearing conditions. Four chickens were randomly selected from each age group at 102 days (Pre), 203 days (Peak), and 394 days (Late), and chickens were euthanized by cervical dislocation after CO_2_ inhalation (inhaled 40%). In the ovaries, mixed samples of small white follicles, large white follicles, and follicular stroma were collected after removal of the follicular fluid. The samples were rapidly frozen by immersion in liquid nitrogen.

### 2.3 RNA extraction

Total RNA from each ovarian tissue was separately extracted using Trizol reagent (Invitrogen, Shanghai solarbio Bioscience & Technology Co., Shanghai, China). Subsequently, the integrity of RNA and DNA contamination were assessed through 1.2% agarose gel electrophoresis. Finally, RNA concentration was determined using the Nanodrop 2000 instrument. Qualified samples were sent to Beijing Novogene Corporation (Beijing, China) for RNA sequencing.

### 2.4 Library construction, sequencing, and transcript assembly

The lncRNA library construction was performed as required, and the library was qualified by Illumina. The raw data (Raw Reads) obtained by the high-throughput sequencing platform Illumina sequencing after quality control and removed from low quality, contaminated, and containing sequences including adaptors (Clean Reads) were used for subsequent analysis. Alignment analysis of the reference genome for the filtered reads using HISAT2 (Pertea et al., 2016). The results of the HISAT2 alignment were spliced using StringTie (Pertea et al., 2015) to obtain the smallest set of transcripts possible, and the transcripts were quantified.

### 2.5 Distribution and identification of LncRNAs

Novel lncRNA classification criteria are as follows: 1) Selection of transcripts containing at least two exons. 2) Transcripts with a length greater than 200 base pairs. 3) Filtering transcripts that overlap with annotated exon regions in the database using Cuffcompare software. Transcripts that overlap with exon regions of annotated spliced transcripts in the database are subsequently annotated as lncRNAs. 4) Retention of transcripts with an expression level calculated as FPKM value greater than or equal to 0.5. 5) Using the Coding-Non-Coding Index (CNCI), Coding Potential Calculator (CPC) (Kong et al., 2007) and Pfam Scan (Pfamsca), we predicted the potential protein-coding ability of each transcript (Sun et al., 2013). During the preprocessing stage, low-quality reads, removal of 3′ adapter/insert tags, elimination of 5′ adapter contaminants and filtering out reads containing poly-A/T/G/C were performed using Illumina Casava (version 1.8) To obtain clean readings from raw data (Finn et al., 2014). Clean reads of lengths between 18 and 30 nucleotides were further filtered for downstream analysis.

### 2.6 Screening for the differentially expressed lncRNAs

Based on the transcriptome splicing results, using the Cuffmerge software to get the combined transcript set, according to the structure of lncRNA and the functional characteristics of encoding protein, set up a series of stringent filtering criteria through five steps: filtering by exon count, transcript length, known transcript annotation, transcript expression, and coding potential. Follow this with cross-analysis using CPC2, CNCI and PFAM for subsequent analysis. Use the StringTie software (Kovaka et al., 2019) to quantify transcripts such as mRNA, lncRNA, and TUCP, obtaining FPKM information for each transcript in each sample. Perform differential analysis using a filtering approach, with threshold criteria set at |log2 (fold change)| > 2 and q-value < 0.05 for selecting differentially expressed transcripts.

### 2.7 Target gene (cis and trans) prediction analysis

There are various mechanisms of lncRNA regulating target genes. In this study, two methods were used to predict lncRNA target genes: 1) co-location: positional correlation target gene analysis, predicting cis target genes according to the positional relationship between lncRNA and mRNA, and the screening range was within 100 k. 2) co-expression: expression correlation target gene analysis predicts the trans target gene according to the expression correlation between lncRNA and mRNA, and the screening conditions are that the absolute value of Pearson correlation coefficient is greater than 0.95 and the *p*-value is < 0.05.

### 2.8 Differential expression analysis of lncRNA target genes, functional enrichment analysis, and the construction of lncRNA-mRNA networks

To study the biological processes of the differentially expressed lncRNAs’ cis- and trans-target genes that were filtered, GO term and KEGG pathway enrichment analyses were conducted. Gene Ontology (GO) is an international standardized classification system for gene functions (Young et al., 2010). GO enrichment analysis was performed on the differentially expressed lncRNAs’ target genes. KEGG (Kyoto Encyclopedia of Genes and Genomes) is a major public database for pathways (Kanehisa et al., 2008) and pathway enrichment analysis, using KOBAS (2.0) with an FDR of 0.05. Based on our previous report on mRNA expression profiles in the same ovarian tissue, differentially expressed genes related to poultry breeding traits and their corresponding specific lncRNAs were selected from the predicted target genes. A lncRNA-mRNA network was constructed using Cytoscape V3.5.1.

### 2.9 Protein-protein interaction analysis

First, we identified lncRNA-regulated mRNAs during different periods. Based on the GO and KEGG pathway enrichment of the mRNAs, we explored the interaction relationships among these genes in the STRING database. Utilizing the extracted relationships from the database, we unveiled the mRNA network and imported the interaction data into Cytoscape software for visualizing the interaction network. This analysis aims to infer the role of lncRNAs in the ovaries.

### 2.10 Validation of lncRNAs and mRNAs by RT-qPCR

Randomly selected six differentially expressed lncRNAs and mRNAs for validation by RT-qPCR, using β-actin as the internal reference. Primer sequences were provided in Table 1. Total cDNA was synthesized using the ReverTra Ace qPCR Master Mix (TOYOBO), qPCR was performed on the 7900 HT Sequence Detection System (ABI, United States). The efficiency of PCR was estimated by four points of serial dilutions of cDNA. The primer concentration in each reaction system was 0.3 μM. The 2^^(-∆∆Ct)^ method was employed to calculate the relative expression levels of the genes. Data obtained were analyzed using GraphPad Prism 3.8. The Student’s t-test (*p < 0.05*) was used for mean comparisons. All results were presented in bar charts.

**TABLE 1:** Primer sequences.

Name	Primer-F	Primer-R	Product length (bp)
LARGE1-OT3	GCA​AGT​CAT​GTA​GAA​GCC​GC	AGG​CTG​AGA​TGC​TTT​GGG​AT	131
LINC5957	CCC​AAG​CAG​AAT​GCT​GAG​AC	TGG​GTG​GGT​GAA​ATG​ACT​GG	124
ENSGALT00000093358	AGC​CGG​ATA​TCT​ACG​GAG​CA	GTG​GTA​TGG​TGC​CTC​TCC​TG	92
LINC8942	TCC​CTC​TGG​GAG​TAC​ATG​GC	TCC​CTC​CAA​GGA​TGT​TGC​CT	136
ENSGALG00000006958-AS3	GAA​GAT​GCC​ACC​GGA​ACC​A	CTC​ACC​TCT​GAA​CGA​GGC​AT	91
LINC7964	TCC​AGG​ACT​GCC​CAT​GAA​AC	TCA​CTG​ACA​ACG​TGG​GAT​GG	104
STMN2	ACG​TCT​CCA​AGA​AAA​GGA​GAG​G	GGG​TCA​CAT​CCA​CCA​TTG​CT	100
GAL	TAC​CTA​CTT​GGG​CCA​CAT​GC	CAT​CAG​CCA​GTG​GTC​TTC​CA	130
INHBB	CTT​CGC​CGA​GAC​AGA​CGA​T	GGC​TGG​CTT​GAA​CGA​CAA​AC	98
EXFABP	CTG​AAC​GAG​ATG​AGG​ACG​CT	ATT​TCC​CTG​CAA​CCT​CGC​TC	109
VCAN	GGA​CAA​AGA​GTT​GAA​CGG​CA	ACA​ACA​TCT​TGA​TCC​CAG​GTT	84
PLAU	ACC​CAA​ATG​GAA​GGA​GCA​GG	GCC​ACA​TGT​ACG​CTC​ACA​CT	101
β-actin	CAG​CCA​GCC​ATG​GAT​GAT​GA	ACC​AAC​CAT​CAC​ACC​CTG​AT	185

## 3 Results

### 3.1 Overview of sequencing data

To identify DE lncRNAs, we analyzed a total of 12 cDNA libraries representing 3 different physiological stages of chicken ovaries, with four biological replicates for each stage. The RNA sequencing generated a total of 224.03 Gb of data. In the sequencing libraries, each sample had an average of 94,062,415 raw reads. After removing low-quality reads and adapter fragments, there were an average of 91,814,733.83 clean reads and 862,827,885.42 mapped reads. The average Q20 content was 96.97%, demonstrating high data quality for Illumina sequencing. Over 92.67% of the clean reads could be accurately mapped to the chicken reference genome. The GC content of the reads from the 12 samples ranged from 89.69% to 91.85%, with a percentage of less than 50% (Table 2). The result indicates that the quality of the sequencing data is sufficiently high to proceed with further analysis.

**TABLE 2:** Summary statistics for sequence quality and alignment information.

Sample	Raw reads	Clean reads	Q20 (%)	Q30 (%)	GC Pct (%)	Total mapped	Uniquely mapped
Late1	91,303,490	89,513,990	96.86	91.59	46.33	84,088,885 (93.94%)	81,637,435 (91.2%)
Late2	101,718,054	99,544,712	96.88	91.76	46.94	93,360,531 (93.79%)	90,319,483 (90.73%)
Late3	89,284,248	87,474,918	96.94	91.77	46.7	82,186,244 (93.95%)	79,133,106 (90.46%)
Late5	88,621,544	86,565,784	96.41	90.51	47.18	80,218,421 (92.67%)	77,715,574 (89.78%)
Pre25	104,413,870	101,823,504	97.07	92.12	46.86	95,666,581 (93.95%)	92,680,183 (91.02%)
Pre26	93,332,334	90,510,302	97.08	92.05	45.56	85,418,005 (94.37%)	83,532,973 (92.29%)
Pre28	90,282,166	88,758,264	97.09	92.09	46.6	83,593,248 (94.18%)	81,077,877 (91.35%)
Pre29	93,306,348	90,256,188	97.03	92	45.51	84,848,360 (94.01%)	82,708,657 (91.64%)
Peak10	92,547,334	90,696,320	97.06	92.09	47.45	85,154,842 (93.89%)	81,684,877 (90.06%)
Peak11	86,443,334	83,522,870	96.88	91.57	46.61	78,938,778 (94.51%)	76,717,770 (91.85%)
Peak12	103,790,790	101,415,404	97.12	92.21	47.15	95,446,885 (94.11%)	91,569,791 (90.29%)
Peak9	93,705,468	91,694,550	97.21	92.38	47.38	86,472,645 (94.31%)	82,238,961 (89.69%)

### 3.2 The identified lncRNAs

**FIGURE 1:**
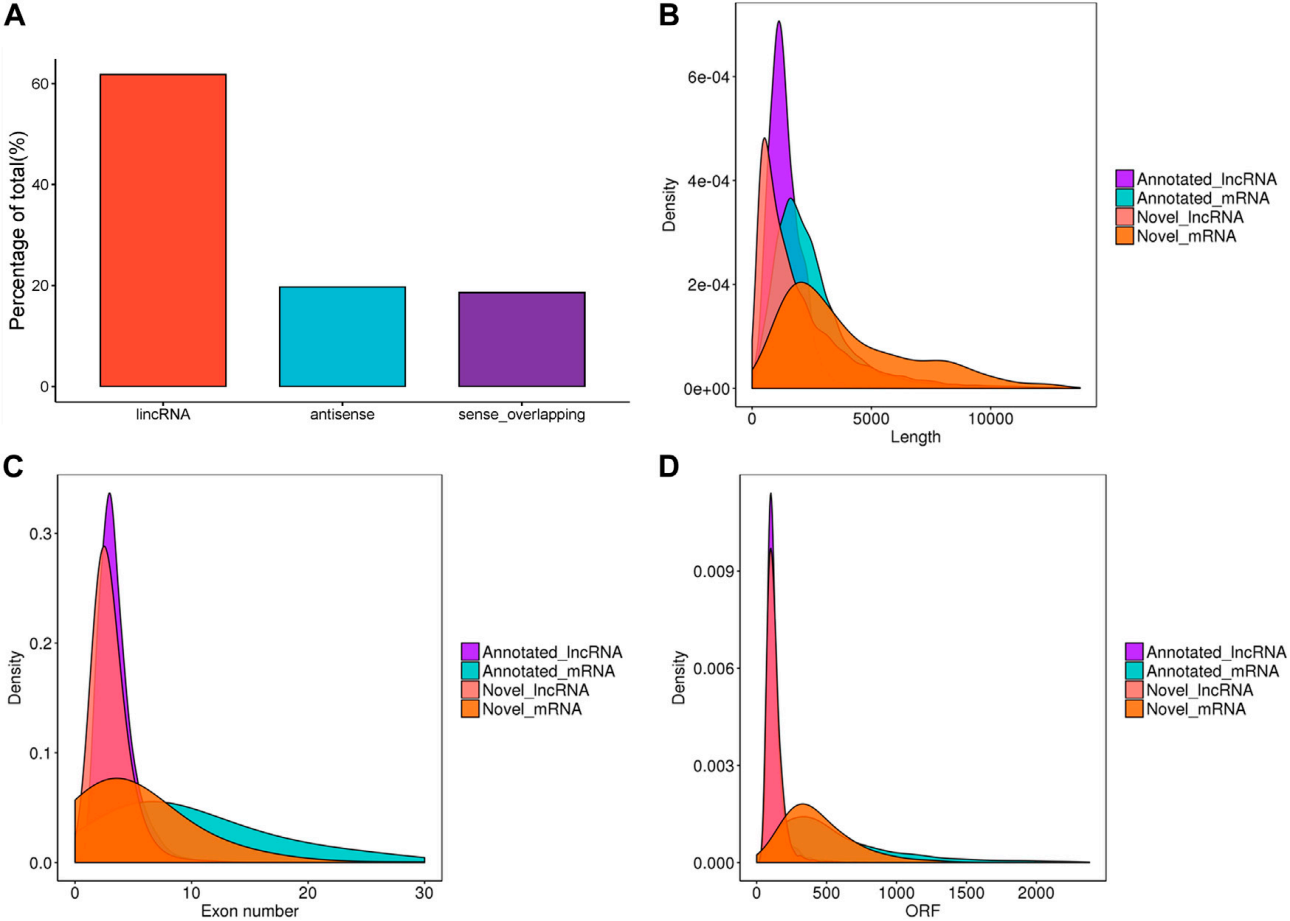
Characteristics of lncRNAs identified in the ovaries of Taihe Black-Bone Chickens. **(A)** Distribution of lncRNA type; **(B)** lncRNA and mRNA transcripts length; **(C)** lncRNA and mRNA exon number; **(D)** lncRNA and mRNA ORF length.

After filtering and potential coding assessment steps, a total of 26,056 candidate lncRNA transcripts were identified, comprising 17,186 known lncRNAs and 8,870 novel lncRNAs. These transcripts were used for further analysis. To gain a deeper understanding of the functional roles of lncRNAs in chicken ovaries, we conducted a genomic background analysis based on their positional relationships with known mRNAs. Among them, we identified 61.8% lincRNA, 19.7% antisense, and 18.6% sense-overlapping (Figure 1A). As shown in Figures 1B–D, the exon number, transcript length, and expression levels of lncRNAs and mRNAs were calculated and plotted. The results showed that the overall trend of lncRNA length was consistent (Figure 1B), most of the exons of lncRNAs were less than 10, significantly lower than that of mRNAs (Figure 1C). The average ORF length of lncRNA was less than that of mRNA (Figure 1D), which suggested that lncRNAs played a crucial role in transcription and post-transcription regulation.

### 3.3 LncRNAs differential expression analysis

**FIGURE 2:**
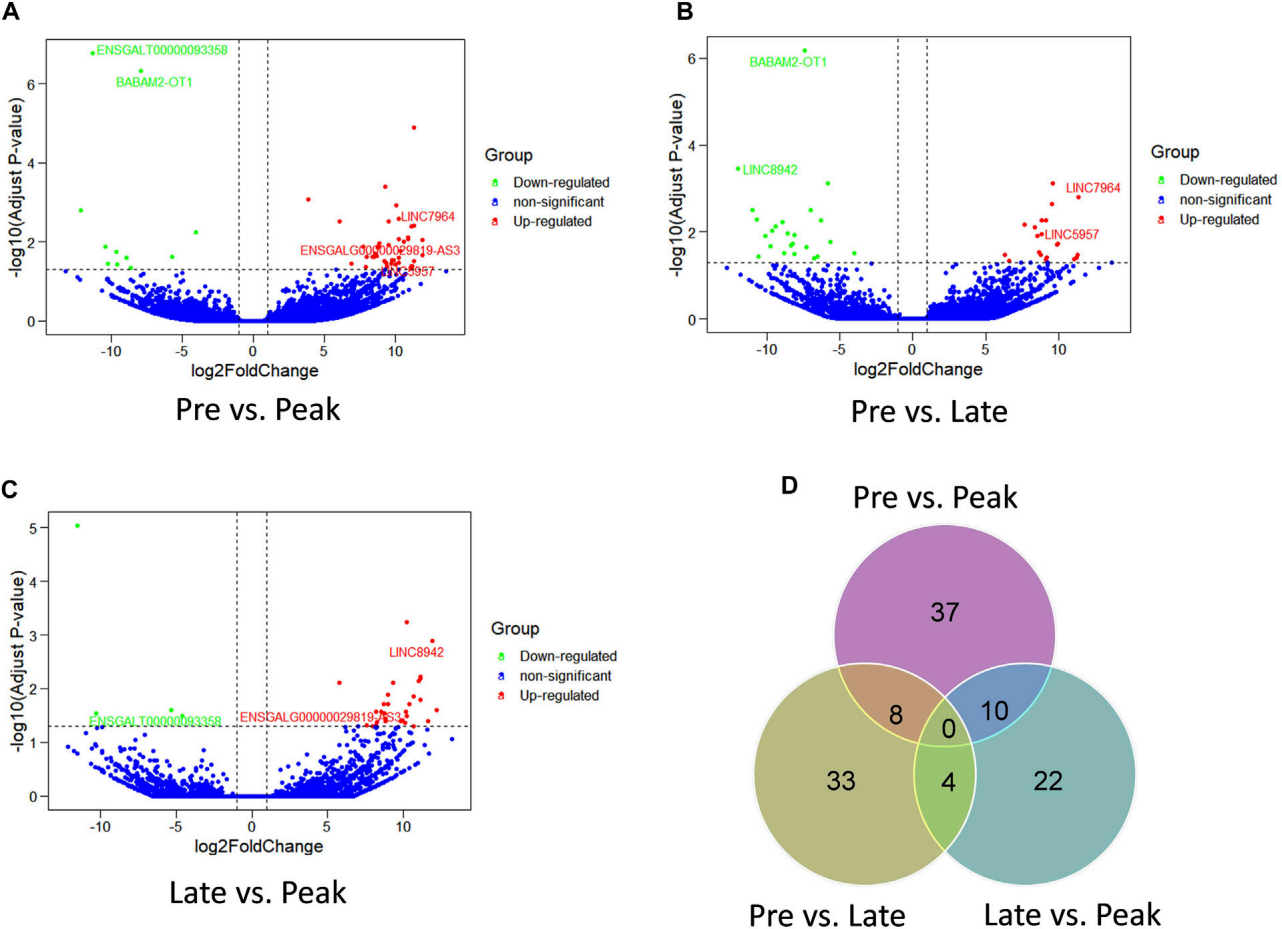
Comparison of expression level and the differential expression of lncRNAs in three groups. **(A)** The volcano plot of lncRNAs in Pre vs. Peak; **(B)** the volcano plot of lncRNAs in Pre vs. Late; **(C)** The volcano plot of lncRNAs in Late vs. Peak. **(D)** Venn diagram of DE lncRNAs of the three groups.

The comparison of lncRNA expression levels were estimated through FPKM. RNA sequencing detected a total of 136 lncRNAs as differentially expressed in the three comparison groups. 55 lncRNAs were differentially expressed (44 upregulated, 11 downregulated) (Figure 2A) in Pre vs. Peak, 45 lncRNAs (21 upregulated, 24 downregulated) in Pre vs. Late (Figure 2B) and 36 lncRNAs (32 upregulated, 4 downregulated) in Late vs. Peak respectively (Figure 2C). To further analyze the interactions between DE lncRNAs, Venn maps were constructed using 55, 45 and 36 lncRNAs differentially expressed in Pre vs. Peak, Pre vs. Late, and Late vs. Peak, respectively (Figure 2D). We did not detect any commonly differentially expressed lncRNAs in the three control groups, but we identified lncRNAs that were specifically differentially expressed in each of the two control groups. Including 8 lncRNAs specifically expressed during the early laying period, 10 lncRNAs specifically expressed during the peak laying period and 4 lncRNAs expressed during the late laying period.

### 3.4 Functional enrichment analysis of differentially expressed lncRNAs target genes

**FIGURE 3:**
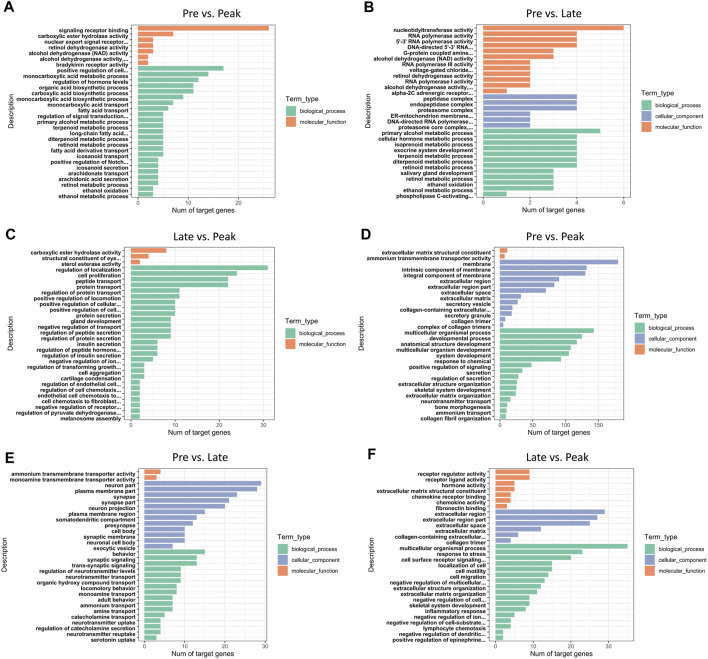
GO annotation of DE lncRNAs’ cis target genes for the top 30 items in **(A)** Pre vs. Peak, **(B)** Pre vs. Late, and **(C)** Late vs. Peak. GO annotation of DE lncRNAs’ trans target genes for the top 30 items in **(D)** Pre vs. Peak, **(E)** Pre vs. Late, and **(F)** Late vs. Peak.

In total, 453 trans-target genes and 445 cis-target genes were identified against the 136 DE lncRNAs. To further interpret the role of particular signaling pathways in ovarian development, we conducted biological functional analysis of the target genes of 136 lncRNAs derived from three control groups. The figures (Figure 3) displayed the top 30 enriched Gene Ontology (GO) items for target genes in each of the three control groups. The GO results for target genes at different laying period suggested significant associations between cellular components, biological processes, and molecular functions with ovarian development and hormone production. During the early laying period, the predominant biological processes included cell signaling, ovarian follicle development, and germ cell development. In terms of molecular functions, there was significant enrichment in receptor ligand activity. Cellular components showed notable enrichment in secretory vesicle, secretory granule, and extracellular region part (Supplementary Table S1). Furthermore, during the peak laying period, GO enrichment projects were similar to the early laying period. However, target genes regulated by lncRNAs specifically expressed during the egg laying peak also involved biological processes such as cell migration, regulation of blood vessel morphogenesis, enzyme-linked receptor protein signaling pathway, and regulation of insulin secretion. Cellular components were significantly enriched in extracellular matrix and collagen trimer (Supplementary Tables S2, S3). In the late laying period, GO items were mainly enriched in processes such as positive regulation of nitrogen compound metabolic process and cellular glucose homeostasis (Supplementary Table S4).

**FIGURE 4:**
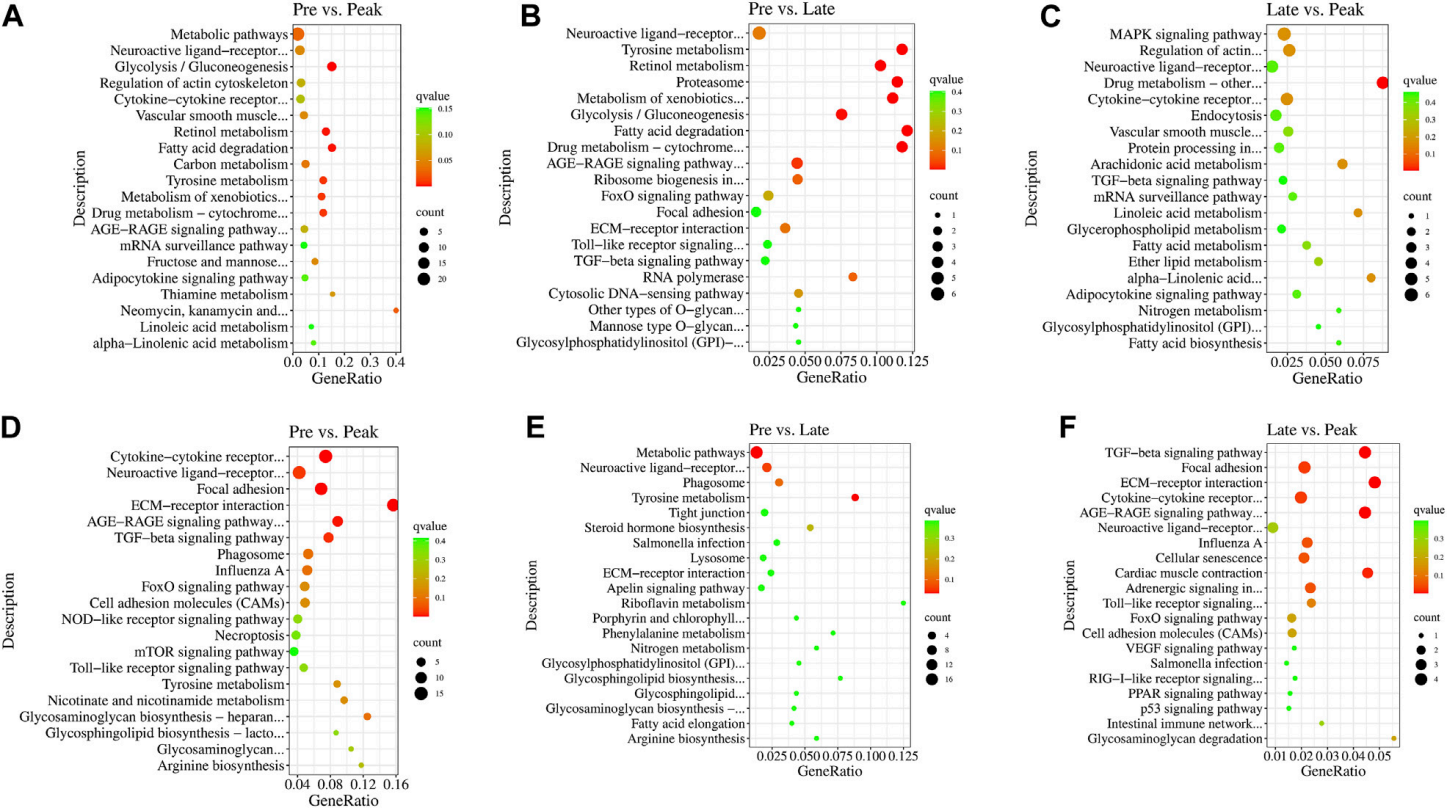
The 20 most significant KEGG pathways of DE lncRNAs’ cis target genes in **(A)** Pre vs. Peak, **(B)** Pre vs. Late, and **(C)** Late vs. Peak. The 20 most significant KEGG pathways of DE lncRNAs’ trans target genes in **(D)** Pre vs. Peak, **(E)** Pre vs. Late, and **(F)** Late vs. Peak.

In the KEGG enrichment analysis, the target genes of DE lncRNAs were significantly enriched in 38 pathways (*p < 0.05*) in Pre vs. Peak, including glycolysis/gluconeogenesis, fatty acid degradation, tyrosine metabolism, and ECM-receptor interaction (Figures 4A, D). In Pre vs. Late, the target genes were significantly enriched to 19 pathways (*p < 0.05*). These included fatty acid degradation, tyrosine metabolism, proteasome and neuroactive ligand-receptor interaction (Figures 4B, E). In Late vs. Peak, they were significantly enriched to 18 pathways (*p < 0.05*), including arachidonic acid metabolism, MAPK signaling pathway, regulation of actin cytoskeleton, and TGF-beta signaling pathway, al (Figures 4C, F). Pathways in the top 20 of the *p*-values are shown in Figure 4. After organizing and analyzing the data, we observed that target genes regulated by various specifically expressed lncRNAs were involved in multiple pathways related to ovary and follicle development. The development of different parts of the ovary is characterized by a series of events influencing sexual maturation. This finding holds significant implications for understanding the function of relevant lncRNAs in the regulation of ovarian development. During the early laying period, enrichment was observed in pathways such as cytokine-cytokine receptor interaction, TGF-beta signaling pathway, and tyrosine metabolism. In the peak laying period, enrichment was observed in pathways in ECM-receptor interaction, focal adhesion, neuroactive ligand-receptor interaction, cell adhesion molecules (CAMs), cytokine-cytokine receptor interaction, and TGF-beta signaling pathway. These pathways align with GO terms related to oocyte cell development. From the results of KEGG pathway enrichment analysis, we infer that lncRNAs may influence these pathways by regulating mRNA during ovarian development processes. The identified pathways are crucial for understanding the intricate regulatory mechanisms involved in the development of the ovary, particularly in relation to oocyte cell development.

### 3.5 LncRNA-mRNA network

**FIGURE 5:**
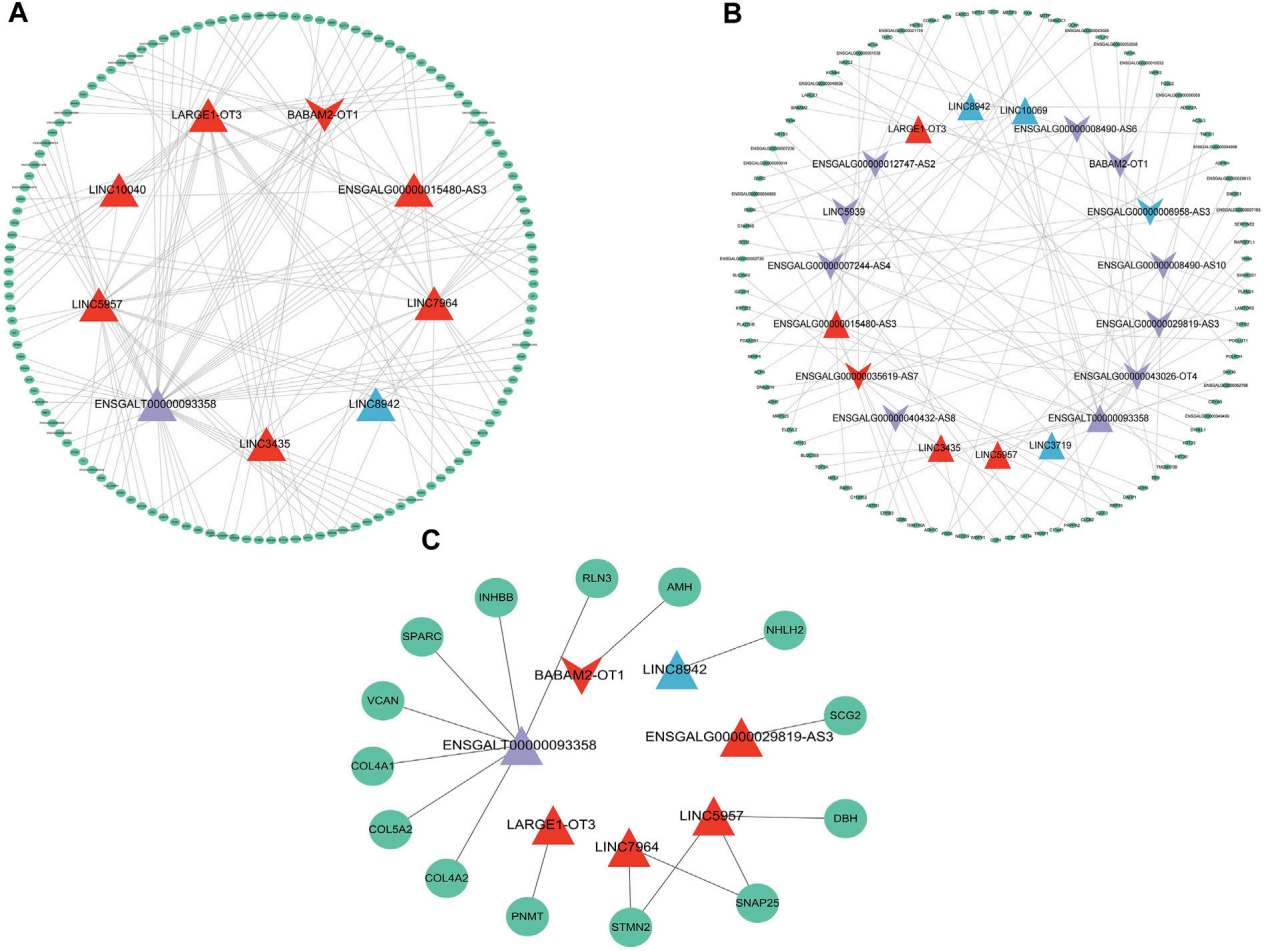
The lncRNA-mRNA interaction network depicting mRNAs regulated by specially expressed lncRNAs with specific expression during various periods. **(A)** mRNAs regulated in trans, **(B)** mRNAs regulated in cis, **(C)** mRNAs related to ovarian development. The triangles and inverted triangle exhibit upregulated and downregulated lncRNAs: red exhibits in the early laying period; purple exhibits during the peak laying period; blue exhibits during the late laying period. The green ellipses exhibit target genes.

Among the 22 lncRNAs specifically expressed during different laying period, we identified that a total of 9 lncRNAs exhibit trans-regulation of the expression of 134 target genes (Figure 5A), while 17 lncRNAs exhibited cis-regulation of 100 target genes (Figure 5B), forming a complex regulatory network. From this, we infer that lncRNAs play a crucial regulatory role in ovarian mRNAs. Through differential analysis and functional analysis of these target genes, we identified 7 lncRNAs that regulated the expression of differentially expressed genes (DEGs) associated with ovarian growth and development. Based on the role of DEGs in ovarian development, we selected a total of 16 lncRNA-mRNA pairs to construct the lncRNA-mRNA network (Figure 5C). During the early laying period, LINC5957 and LINC7964 jointly trans-regulated the expression levels of stathmin-like 2 (*STMN2*), synaptosome-associated protein 25 (*SNAP25*), and LINC5957 also trans-regulated Dopamine β-hydroxylase (*DBH*). BABAM2-OT1 trans-regulated anti-Müllerian hormone (*AMH*). LARGE1-OT3 trans-regulated phenylethanolamine N-methyltransferase (*PNMT*). These target genes were enriched in cytokine-cytokine receptor interaction, TGF-beta signaling pathway, and tyrosine metabolism, and participate in follicle development. During the peak laying period, ENSGALT00000093358 trans-regulated the expression levels of inhibin subunit beta B(INHBB), relaxin-3 (*RLN3*), versican (*VCAN*), secretogranin II (*SCG2*), secreted protein, acidic and rich in cysteine (*SPARC*), collagen family members *COL5A2*, *COL4A1*, and *COL4A2*. The downregulated ENSGALG00000029819-AS3 positively regulated the expression level of secretogranin II (*SCG2*). These target genes were involved in oocyte cell development, oogenesis, ECM-receptor interaction, focal adhesion, neuroactive ligand-receptor interaction, CAMs, cytokine-cytokine receptor interaction, and TGF-beta signaling pathway. During the late laying period, LINC8942 trans-regulated the expression of *NHLH2*. It is evident that these lncRNAs participate in the regulation of ovarian growth and development by targeting mRNAs.

### 3.6 Protein–protein interaction between mRNAs regulated by lncRNAs with temporal specificity in different periods

**FIGURE 6:**
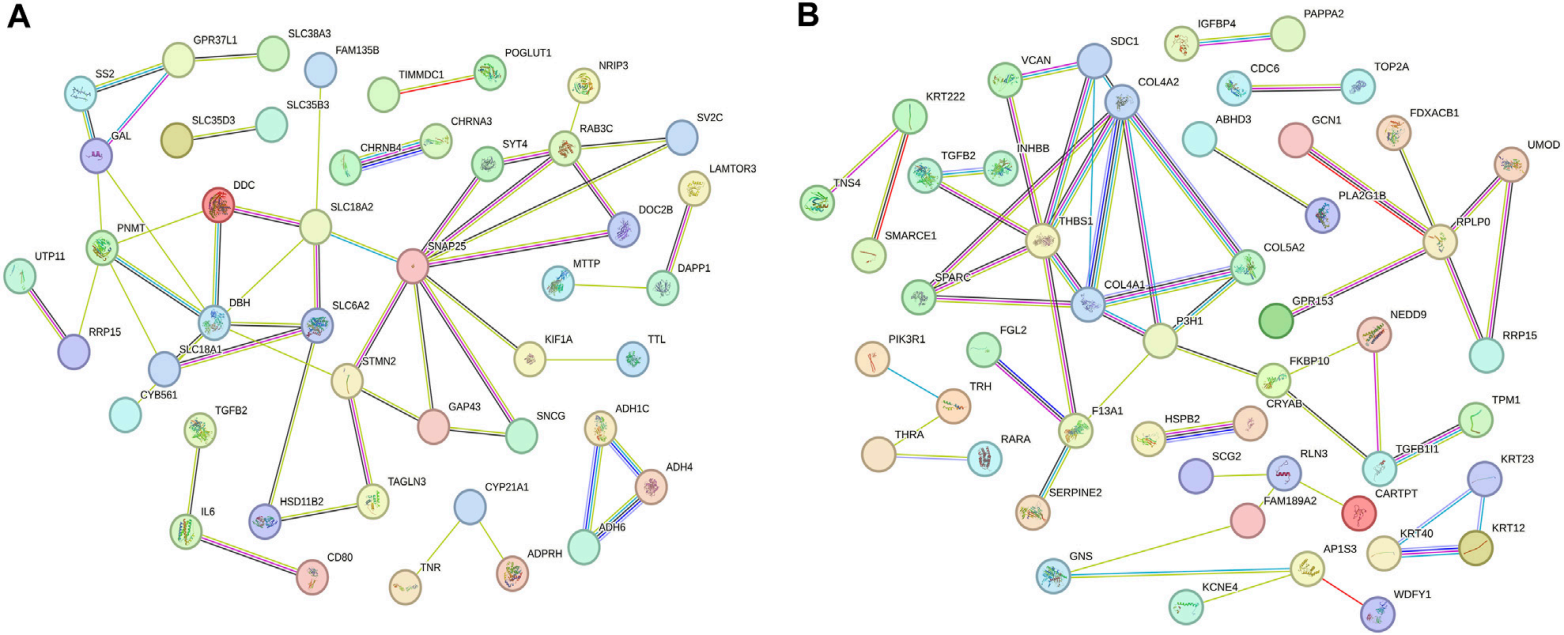
Protein–protein interaction network of mRNAs regulated by lncRNAs specifically expressed across various periods. **(A)** The early laying period, **(B)** the peak laying period.

Based on the enrichment analysis of GO and KEGG pathways for target genes, we conducted a protein-protein interaction network analysis for mRNAs regulated by lncRNAs with specific expression during different laying periods. This approach provides insight into potential functional networks of mRNAs that regulate ovarian growth and development. We identified key genes, such as *SNAP25*, *STMN2*, *DBH*, and *PNMT*, as central hub genes in the protein-protein interaction network during the early laying period (highly correlated in the candidate module, with the top 40% connectivity, Figure 6A). Similarly, during the peak laying period, *COL4A2*, *COL4A1*, *COL5A2*, *SPARC*, and other key genes were identified as central hub genes in the protein-protein interaction network (highly correlated in the candidate module, with the top 40% connectivity, Figure 6B). Previous studies have reported the crucial roles of these genes in regulating ovarian growth and development. It is noteworthy that these hub genes exhibit differential expression during different laying periods, suggesting that they may play distinct roles at different periods of ovarian development. These findings provide clues for a more in-depth understanding of the precise roles of these key genes in the ovarian development process and offer important directions for future research.

**FIGURE 7:**
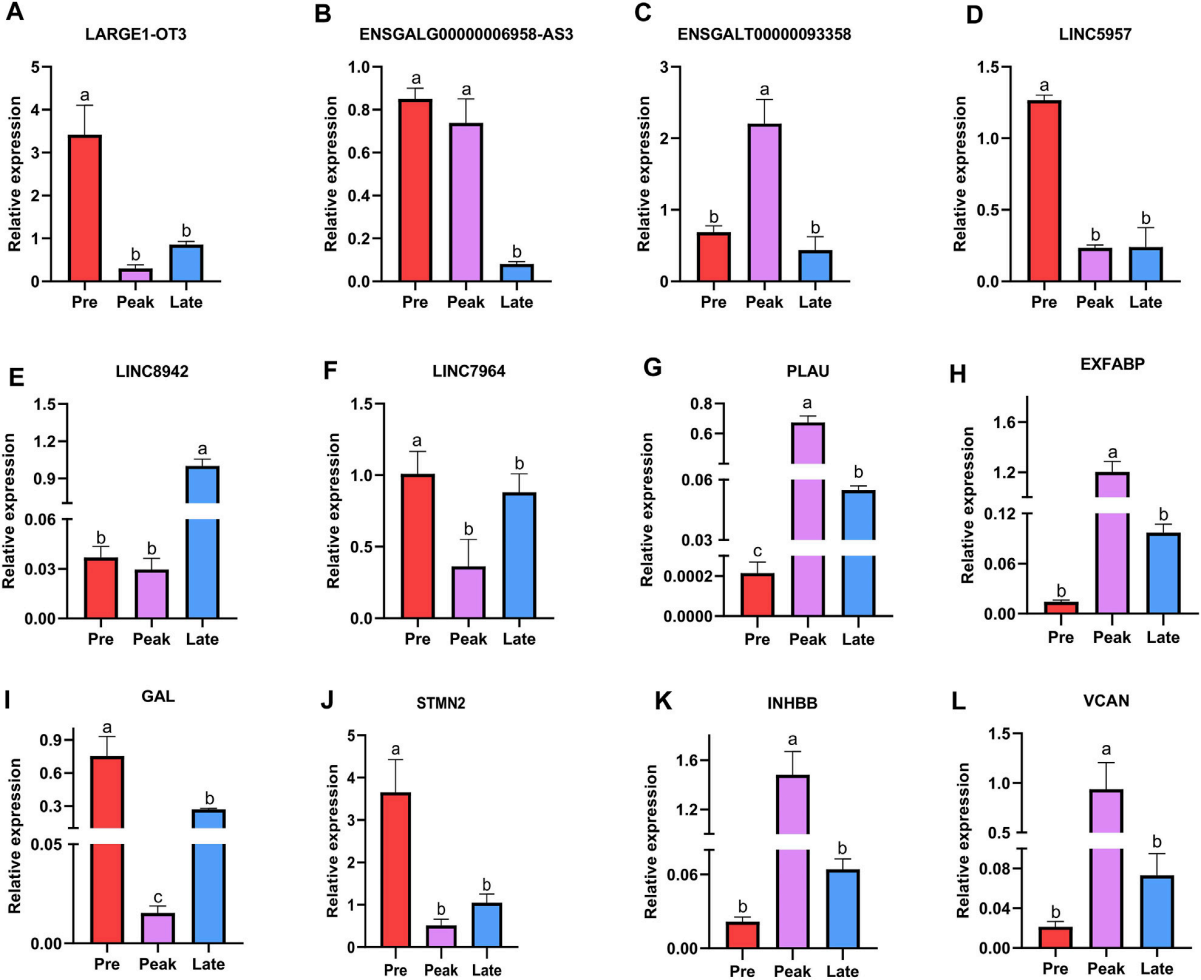
Validation of six differentially expressed lncRNAs and six differentially expressed mRNAs was performed by qPCR. The letters on the column diagram indicate significant (*p* < 0.05) differences at different stages. **(A–F)** represents lncRNA, and **(G–L)** represents mRNA.

Finally, we conducted RT-qPCR analysis to assess the expression levels of the six lncRNAs and six mRNAs across various laying periods. As depicted in Figure 7, the qPCR expression results aligned with the trends observed in the RNA-seq data, offering valuable insights for the potential functional validation of these molecules in our subsequent experiments.

## 4 Discussion

Egg production is a crucial indicator for assessing poultry fertility, and it significantly influences the production efficiency and profitability of the layer chicken industry (Mu et al., 2021). The laying process is governed by ovarian function and is stimulated by specific peptides or hormones secreted by the HPG axis, promoting the maturation and ovulation of follicles (Tilly and Johnson, 1990; Onagbesan et al., 2009). In recent years, an increasing amount of research has shown that lncRNAs play a significant role in wide-ranging biological processes (Liu et al., 2018). Researchers have found that lncRNAs participate in various reproductive processes in animals. Including pregnancy (Nakagawa et al., 2014), gonadotropin responses (Li et al., 2013), oocyte maturation (Li et al., 2015), and placental formation (Gao et al., 2012). In order to gain a better understanding of the physiological characteristics of Taihe Black-Bone Chickens and enhance their reproductive capabilities, this study conducted an in-depth analysis of the expression patterns of ovarian lncRNAs during the developmental process. Therefore, this study constructed 12 ovarian cDNA libraries from three different laying periods and assessed the expression of lncRNAs using Illumina high-throughput sequencing. The aim is to study functional lncRNAs associated with egg laying characteristics.

This study marks the first report of the expression profile of lncRNAs in the ovaries of Taihe Black-Bone Chickens. Specifically, we identified a total of 136 differentially expressed lncRNAs across three distinct laying periods, uncovering lncRNAs that exhibited stage-specific expression patterns (Figure 2). When comparing the characteristics of lncRNAs with mRNAs, we observed that lncRNAs had fewer exons, transcripts, and shorter open reading frame lengths, and their abundance was lower than that of mRNAs, consistent with findings in other species (Tilgner et al., 2012). The lncRNAs identified in this study exhibited similar characteristics to those found in previous research, indicating the reliability of their detection in this study.

**FIGURE 8:**
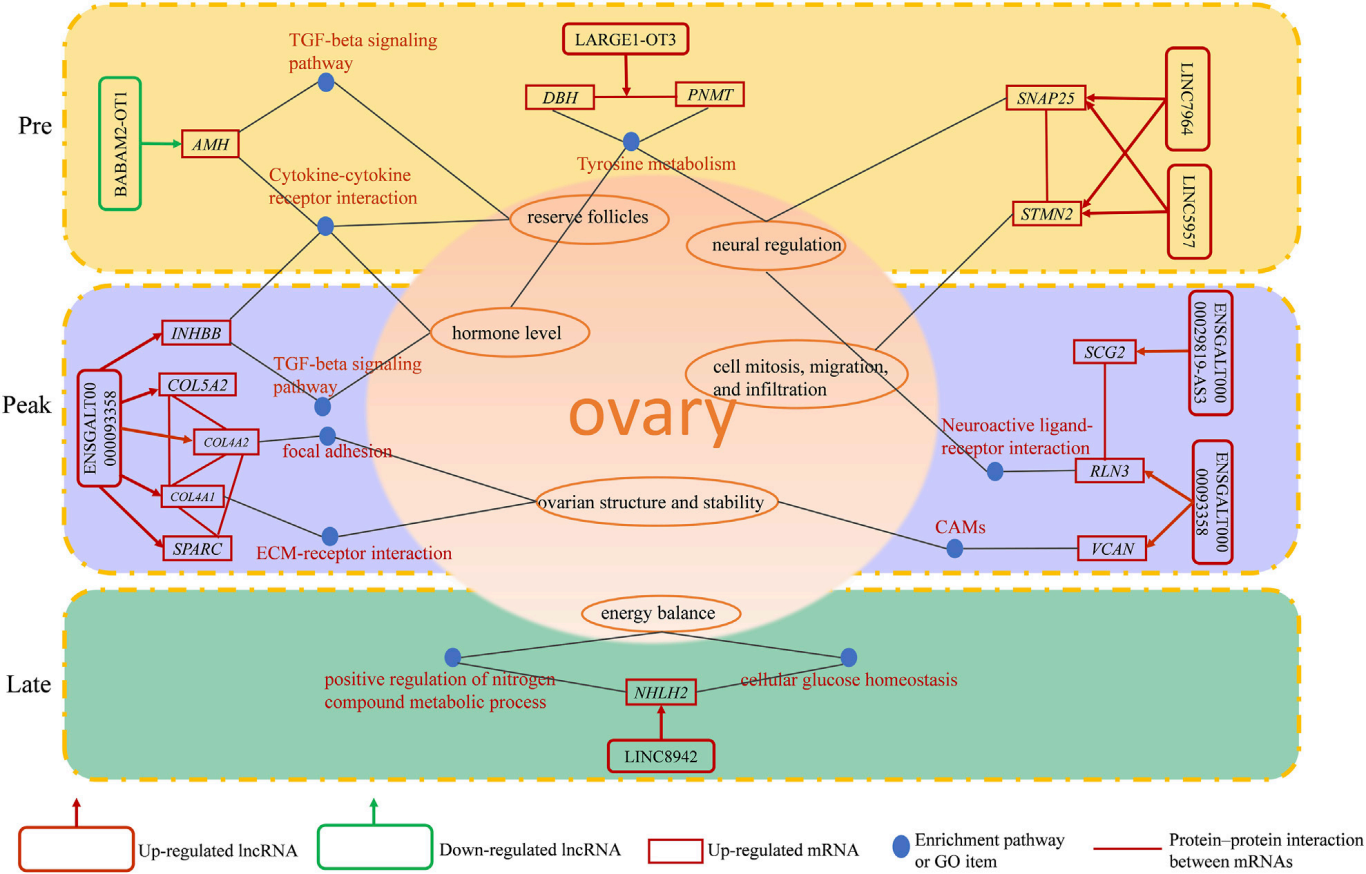
A schematic diagram of the regulatory role of identified lncRNAs in ovarian function.

In this study, we observed some lncRNAs with stage-specific expression patterns regulated the expression of target genes related to ovarian development (Figure 8). In the GO analysis during the early laying period, these target genes were enriched in items such as follicle development, germ cell development, and hormone synthesis (Supplementary Table S1). In the KEGG analysis, the target genes were predominantly enriched in the pathways of Cytokine-cytokine receptor interaction, TGF-beta signaling pathway, and Tyrosine metabolism (Figures 4D, E). Cytokine-cytokine receptor interaction is a biological pathway involved in regulating immune and inflammatory processes. It plays a role in immune cell regulation in ovarian tissue to ensure a normal immune response. This pathway is also related to the regulation of ovarian hormones, affecting processes like follicle development, egg maturation, and ovulation. TGF-beta signaling pathway plays a crucial role in the proliferation, differentiation, and survival of granulosa cells and follicular wall cells (Han et al., 2022). Additionally, it regulates the selection and release of follicles. The target gene *AMH* is significantly enriched in both pathways. Anti-Müllerian hormone (*AMH*) belongs to the transforming growth factor-beta super family and is mainly secreted by granulosa cells. It inhibits the activation of primordial follicles in the early stages of ovarian follicle development, preventing the depletion of the follicle pool. Research indicates that during the follicle selection stage, *AMH* significantly decreases (Xu J. et al., 2016). Additionally, different concentrations of *AMH* treatment have varying effects on follicular development and steroidogenesis in the reproductive organs of laying hens (Huang et al., 2021). *AMH* exhibited high specific expression in the early laying period (Supplementary Tables S5, S6) and was inversely regulated by downregulated BABAM2-OT1. This regulation contributes to maintaining the reserve of follicles and preventing their premature development.

Tyrosine metabolism plays an essential role in ovarian function and physiological processes, especially in hormone synthesis, neural regulation, and antioxidant defense. Tyrosine is a crucial precursor molecule for hormone biosynthesis. In the ovaries, tyrosine is involved in the synthesis of progesterone and catecholamine hormones. The target genes enriched in this pathway include *PNMT* and *DBH*. Dopamine β-hydroxylase (*DBH*) and phenylethanolamine N-methyltransferase (*PNMT*) are key enzymes in the biosynthesis of catecholamines, with *PNMT* being responsible for a crucial step in the biosynthesis of catecholamine neurotransmitters. Its primary function is to convert the precursor molecule phenylethanolamine into norepinephrine. Moreover, studies suggest that *DBH* regulates reproductive activity in geese through the HPG axis. In the ovaries of high-egg-producing Yangzhou geese, the expression level of *DBH* is higher than in low-egg-producing counterparts. It is speculated that a higher egg production requires the release of more hormones for ovulation, and a higher expression level of *DBH* ensures an adequate hormone secretion (Xu Q. et al., 2016). The upregulated LARGE1-OT3 inversely regulated *PNMT*, *PNMT* was highly expressed in the early laying period of the ovaries (Supplementary Tables S5, S6), while *DBH* was upregulated in Pre vs. Late (Supplementary Table S6) and was inversely regulated by the upregulated LINC5957. It is evident that LARGE1-OT3 and LINC5957, by regulating the expression of *PNMT* and *DBH*, contribute to maintaining neurotransmitter balance and hormone levels in the early laying period of the ovaries.

Furthermore, the specific high expression of *STMN2* and *SNAP25* during the early laying period has captured our attention (Supplementary Tables S5, S6). These two target genes were hub genes in protein-protein interaction networks (Figure 6A), and we infer that they play crucial roles in the ovaries of Taihe Black-bone Chickens during the early laying period. Stathmin 2 (*STMN2*) is a gene encoding a member of the stathmin family. Stathmins are a protein family that regulates the dynamic stability of microtubules, influencing ovarian cell mitosis, migration, and infiltration, and regulating intracellular signal transduction. Reports suggest that the expression of *STMN2* in the hypothalamus and pituitary of native Taiwanese chickens influences the number of eggs at 50 weeks of age and the laying rate after the first egg (Chen et al., 2007). These findings emphasize the significant role of *STMN2* in the chicken ovaries. Synaptosome-associated protein 25 (*SNAP25*) is one of the key proteins involved in the fusion of neuronal synaptic vesicles, participating in the process of neurotransmitter release (Shimada et al., 2007). It may be involved in neurotransmitter release in ovarian tissue, regulating cell communication and signal transduction within the ovaries. The upregulated LINC5957 and LINC7964 collectively inversely regulated *STMN2* and *SNAP25*, impacting the overall functionality of ovarian tissue.

During the peak laying period, ENSGALT00000093358 was the most upregulated specific lncRNA. It inversely regulated the expression of *INHBB*, *RLN3*, *VCAN*, *COL4A1*, *COL4A2*, *COL5A2*, and *SPARC*, while the downregulated ENSGALG00000029819-AS3 positively regulated the expression level of *SCG2*. These target genes were involved in biological processes such as ovarian cell migration, regulation of vascular morphogenesis, enzyme-linked receptor protein signaling pathway, insulin secretion regulation, as well as cellular components like follicular wall extracellular matrix and collagen trimer, according to the GO analysis (Supplementary Tables S2, S3). In KEGG analysis of the target genes, we found that ECM-receptor interaction is a crucial cell signaling pathway, consistent with the results of the GO analysis. As known, the ovarian follicular wall of hens mainly consists of the extracellular matrix (ECM), and the ability of the ECM to guide cell proliferation, differentiation, and function highlights its critical role in normal ovarian function reconstruction. Focal adhesion is a cellular structure that connects cells to the ECM, achieved by the binding of receptors on the cell membrane to the ECM (Huang et al., 2022). ECM-receptor interaction may participate in the interaction between granulosa cells and the follicular wall, influencing the formation and maturation of follicles. *COL4A1* and *COL4A2*, two crucial genes, are enriched in these pathways. *COL4A1* and *COL4A2* encode collagen proteins (collagen type IV alpha 1 chain and collagen type IV alpha 2 chain, respectively). In the ovaries, these genes are involved in the structure and function of the extracellular matrix (ECM) and interactions with cells (Tang et al., 2019). *COL4A1* was specifically highly expressed during the high-production period, and *COL4A2* and *COL5A2* were upregulated in Peak vs. Pre (Supplementary Table S5). Additionally, upregulated secreted protein, acidic and rich in cysteine (*SPARC*) in Peak vs. Pre (Supplementary Table S5), was a hub gene in the protein-protein interaction network during the peak laying period and is involved in the regulation of the extracellular matrix (Figure 6B; Supplementary Table S2). Therefore, ENSGALT00000093358 may maintain ovarian structure and stability by regulating these target genes.

Neuroactive ligand-receptor interaction is a biological pathway involving the interaction between neurotransmitters and their receptors. There are neurons present in ovarian tissue that may release neurotransmitters, interacting with corresponding receptors and influencing hormone secretion and physiological effects in the ovary, participating in reproductive regulation. *RLN3* is significantly enriched in this pathway and is highly expressed during the peak egg-laying period. Relaxin-like peptides, such as *RLN3*, are produced in granulosa cells post-ovulation and play a role in promoting egg laying, impacting the oviduct and ovary (Ghanem and Johnson, 2021). Additionally, in the protein-protein interaction network, we observed an interaction between *RLN3* and *SCG2* (Figure 6B). Secretogranin II (*SCG2*) is a protein involved in neurosecretion, particularly associated with the secretion granules of neuroendocrine cells. *SCG2* was upregulated in Peak vs. Late (Supplementary Table S7). In the ovaries of Taihe Black-bone Chickens, these two target genes may influence ovarian function through neural regulation, affecting the laying rate during the peak laying period. CAMs are glycoproteins on the cell membrane that facilitate adhesion of different types of ovarian cells, coordinating interactions between oocytes and supporting cells within the follicle (Heffner et al., 2020). Versican (*VCAN*) was significantly enriched in CAMs and was highly expressed during the peak laying period, promoting follicle maturation. *INHBB* (Inhibin subunit beta B) is a glycoprotein hormone belonging to the transforming growth factor-beta superfamily, known to simultaneously affect apoptosis and steroidogenesis in primary granulosa cells (M'Baye et al., 2015). High expression of *INHBB* during the peak laying period (Supplementary Tables S5, S7), along with its enrichment in the Cytokine-cytokine receptor interaction and TGF-beta signaling pathway, suggests its role in regulating hormone secretion and maintaining reproductive system balance through a feedback mechanism. Therefore, based on the functional analysis of target genes, it is inferred that during the peak laying period, the lncRNAs ENSGALT00000093358 and ENSGALG00000029819-AS3, which show specific expression, may play crucial regulatory roles in target genes associated with ovarian development, participating in the regulation of ovarian follicle development and maturation.

In our analysis of the late laying period ovarian-specific lncRNAs expression, the upregulation of LINC8942 caught our attention as it was found to exhibit trans regulation of *NHLH2* expression. *NHLH2* is a member of the basic helix-loop-helix (*bHLH*) transcription factor family. The GO items of this target gene was significantly enriched in positive regulation of nitrogen compound metabolic process and cellular glucose homeostasis (Supplementary Table S4), suggesting that *NHLH2* participates in the regulation of ovarian glucose metabolism and energy balance. In addition, in a study on mouse gonadal development, *NHLH2* was found to be necessary for the migration of embryonic GnRH neurons, and migration defects could potentially impact gonadal development (Good and Braun, 2013). This target gene was downregulated in Pre vs. Late (Supplementary Table S6). Consequently, it can be inferred that LINC8942 may influence the late laying period egg production by regulating genes associated with gonadal development and energy metabolism. Despite our observations, the underlying mechanisms need further investigation

## 5 Conclusion

In this study, we conducted RNA-seq analysis on Taihe Black-Bone Chicken ovaries at different laying periods. We identified 136 DE lncRNAs. Among these, 8 were specific to early laying periods, 10 to peak laying periods, and 4 to late laying periods. Seven stage-specific DE lncRNAs regulated 14 target genes associated with ovarian development. During the early laying period, upregulated lncRNAs (LINC5957, LINC7964) co-trans-regulated *STMN2*, *SNAP25*, and cis-regulated *DBH*. LARGE1-OT3 cis-regulated *PNMT*. The downregulated DE lncRNA BABAM2-OT1 trans-regulated *AMH*. Upregulated ENSGALT00000093358 trans-regulated *INHBB*, *RLN3*, *VCAN*, *SPARC*, *COL5A2*, *COL4A1*, *COL4A2*, while upregulated ENSGALG00000029819-AS3 cis-regulated *SCG2* in the peak laying period. LINC8942 trans-regulated *NHLH2* in the late laying period. These target genes are involved in follicular development, oocyte-related signaling pathways (tyrosine metabolism, CAMs, neuroactive ligand-receptor interactions, focal adhesion, cytokine-cytokine receptor interactions, TGF-beta signaling pathway). This study provides a foundation for further research on the impact of lncRNAs on reproductive characteristics of Taihe Black-Bone Chickens. Future studies can explore functional roles of these lncRNAs and use genetic modification to enhance chicken reproductive performance and ovarian health. Comparative analysis with other species can yield insights into avian reproductive biology.

## Data Availability

The sequence data were submitted to the NCBI SRA database under the accession number PRJNA889190.
